# Differences in do-not-resuscitate orders, hospice care utilization, and late referral to hospice care between cancer and non-cancer decedents in a tertiary Hospital in Taiwan between 2010 and 2015: a hospital-based observational study

**DOI:** 10.1186/s12904-018-0271-y

**Published:** 2018-01-24

**Authors:** Tzu-Chien Shih, Hsiao-Ting Chang, Ming-Hwai Lin, Chun-Ku Chen, Tzeng-Ji Chen, Shinn-Jang Hwang

**Affiliations:** 1 Department of Family Medicine, Taoyuan General Hospital, No. 1492, Zhongshan Rd., Taoyuan Dist, Taoyuan City, 330 Taiwan, Republic of China; 2 Department of Family Medicine, Taipei Veterans General Hospital, No. 201, Sec. 2, Shipai Rd., Beitou Dist, Taipei City, 11217 Taiwan, Republic of China; 3 School of Medicine, National Yang-Ming University, No. 155, Sec. 2, Linong Street, Taipei City, 11221 Taiwan, Republic of China; 4 Department of Radiology, Taipei Veterans General Hospital, No. 201, Sec. 2, Shipai Rd., Beitou Dist, Taipei City, 11217 Taiwan, Republic of China; 5 Institute of Clinical Medicine, National Yang-Ming University, No. 155, Sec. 2, Linong Street, Taipei City, 11221 Taiwan, Republic of China

**Keywords:** Hospice care utilization, Do-not-resuscitate order, Terminal cancer patients, Non-cancer disease, Late referral

## Abstract

**Background:**

In 2009, the Taiwanese national health insurance system substantially expanded hospice coverage for terminal cancer patients to include patients with end-stage brain, dementia, heart, lung, liver, and kidney diseases. This study aimed to evaluate differences in do-not-resuscitate (DNR) status and hospice care utilization between terminal cancer patients and advanced non-cancer patients after the policy change.

**Methods:**

Data were obtained from the Death and Hospice Palliative Care Database of Taipei Veterans General Hospital in Taiwan. The differences between cancer and non-cancer patients who died in this hospital between 2010 and 2015 were analyzed in terms of patient characteristics, rates of DNR orders, hospice care utilization, number of living days after DNR order, duration of survival (DOS) after hospice care enrollment, and the rate of late referral to hospice care.

**Results:**

Data for 8459 patients who died of cancer and major non-cancer terminal diseases were included. DNR order rate, hospice care utilization rate, and DOS were significantly higher for cancer patients than for non-cancer patients (*p* < 0.001, *p* < 0.001, and *p* < 0.001, respectively). The number of living days after DNR order and the late referral rate were significantly higher for non-cancer decedents than for cancer decedents (*p* < 0.001 and *p* < 0.001, respectively). From 2010 to 2015, there were significantly increasing trends in the hospice utilization rate, number of living days after DNR order, and rate of late referral for the cancer group (*p* < 0.001, *p* = 0.001, and *p* < 0.001, respectively). For the non-cancer group, there were significantly increasing trends in the rate of DNR order, hospice utilization rate, and number of living days after DNR order (*p* < 0.001, *p* < 0.001, and *p* = 0.029, respectively).

**Conclusions:**

Further guidelines should be developed to help clinicians to promptly refer terminal cancer and non-cancer patients to hospice care. Considering the lower hospice utilization rate and the growing need for hospice care among terminal non-cancer patients, policymakers should consider how to improve the relevant levels of professional care to enhance the accessibility and availability of hospice care in Taiwan.

## Background

It is well-known that cancer patients and non-cancer patients living with advanced/terminal illness may suffer from similar symptom burdens [1–4]. To avoid needless suffering and even harmful resuscitation, patients with an advanced/terminal illness can choose to sign a do-not-resuscitate (DNR) order when they are critically ill [5]. Such patients can also choose to receive hospice care to treat their symptoms and improve their quality of life in their final days [6–8].

However, little attention has been paid to the symptom management needs of patients with life-threatening diseases other than cancer [2, 4]. In Germany, non-cancer patients accounted for only 8.1% of all the patients who received specialist palliative care from 2007 to 2011 [9]. Only 11% of non-cancer patients in the UK were treated in hospice specialist inpatient care units from 2010 to 2011 [10]. In contrast, the percentage of non-cancer patients receiving hospice care in the USA is much higher (62% in 2012) [11]. Despite these large international differences in the proportion of different types of patients in hospice care systems, there are no studies comparing rates of hospice care utilization between cancer and non-cancer patients in Asia. Therefore, there is a pressing need for comparisons of the rates of hospice care provided to both cancer and non-cancer patients in Asian countries.

Duration of survival (DOS) after hospice care enrollment is an important outcome indicator in end-of-life care [12]. Previous studies show that the DOS after hospice care enrollment is shorter in patients with heart failure than in cancer patients [7, 13, 14]. These studies found that disease prognoses and disease trajectories for patients with heart failure and cancer were major factors in determining the timing of hospice care enrollment and referral. However, investigations are needed of hospice care utilization in other major non-cancer diseases.

Since 1995, the Department of Health in Taiwan has been proactive in the development of hospice care programs [12]. The Taiwanese national health insurance (NHI) system started to provide coverage for hospice home care in 1996, hospice inpatient care in 2000, and hospice shared care (including hospice specialist consultations and nursing care) in 2004. However, these hospice care programs initially only provided care for terminal cancer patients. In 2003, patients with motor neuron disease (amyotrophic lateral sclerosis) were able to receive hospice care. In 2009, the NHI system expanded hospice care coverage to include patients with end-stage brain diseases, dementia, heart diseases, lung diseases, liver diseases, and renal diseases [15, 16]. Taiwan’s NHI system currently covers hospice care of various types, including hospice inpatient services, hospice shared care, and hospice home care for patients with advanced cancer and the above-mentioned terminal non-cancer diseases.

Investigation of hospice utilization by non-cancer patients has been neglected [2, 4]. Although previous studies have reported a trend toward late referrals to hospice care for cancer patients, the issue of late referrals for non-cancer patients has not been sufficiently examined. To address this research gap, this study aimed to evaluate the differences between cancer patients and non-cancer patients who had died in a tertiary hospital in Taiwan between 2010 and 2015. The investigation focused on differences in patient characteristics, the rate of DNR orders, hospice care utilization, number of living days after DNR order, DOS after hospice care enrollment, and the rate of late referral to hospice care.

## Methods

### Data source

Data for this cross-sectional study were obtained from the Death and Hospice Palliative Care Database (DHPCD) of Taipei Veterans General Hospital in Taiwan. Data for patients 20 years old and over who had died of cancer or major non-cancer terminal diseases (including terminal brain diseases, motor neuron disease, dementia, heart failure, terminal lung diseases, liver failure, and renal failure) in the hospital from 2010 through 2015 were included. Patients’ age at death, gender, major diagnosis, date of the last admission, DNR order status, date of DNR declaration, hospice care status, date of hospice care enrollment, and date of death were extracted from the DHPCD.

Taipei Veterans General Hospital is a tertiary hospital located in an urban area in Taiwan and has 2941 licensed beds. The hospice care team was founded in 1997. To provide comprehensive care, the team includes physicians, nurses, clinical psychotherapists, social workers, spiritual therapists, and art therapists. The hospice care team provides hospice inpatient care in the hospice ward; hospice shared care for patients admitted to ordinary wards in need of hospice care; and hospice home care for patients living at home, in the community, or in institutions [16].

The data for the living days after DNR order show the number of days of survival after a patient had signed the DNR consent. The DOS after hospice enrollment, which represented the length of stay in hospice care, was defined as the interval between the date of first enrollment in hospice care and the date of death. Late referral was defined as a DOS after hospice enrollment of less than 7 days [17]. DNR order rate (in percent) was calculated as the number of patients who signed a DNR order divided by the number of patients who died; hospice care utilization rate (in percent) was calculated as the number of patients who received hospice care divided by the number of patients who died.

### Statistical analysis

Statistical analyses were performed using IBM SPSS version 22.0 (IBM Corporation, Armonk, NY, USA). Gender distributions, late referrals, DNR rates, and hospice utilization rates were analyzed using chi-square tests. Age, length of hospital stay, number of living days after DNR order, and DOS after hospice enrollment were analyzed using the Mann–Whitney U test. As the number of patients who signed a DNR order and the number of patients who received hospice care during the study period were ordinal independent variables (and not normally distributed), trend analyses were conducted using the non-parametric Jonckheere–Terpstra test. A two-tailed *p* value < 0.05 was considered statistically significant.

## Results

### Patient characteristics

**Fig. 1:**
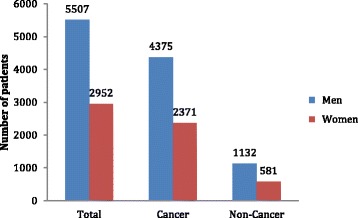
Distributions of cancer and non-cancer decedents by gender

Data for 8459 patients were included in the study. Of these patients, 6746 died of cancer and 1713 died of major non-cancer diseases from 2010 to 2015. Most of these decedents were male (*n* = 5507, 65.1%) (Fig. 1). The median age was 72 years (interquartile range [IQR]: 24; range: 20–103 years). The median length of hospital stay was 16 days (IQR: 23 days; range: 1–869 days). Gender distribution and length of hospital stay were not significantly different between cancer and non-cancer patients (*p* = 0.349 and 0.529, respectively). However, cancer patients were significantly younger than non-cancer patients (median age of 70 years vs. 81 years, *p* < 0.001) (Table 1).

**Table 1:** Characteristics of patients

Characteristics	Total	Cancer	Non-Cancer	*p*
Gender, *n* (%)				
Men	5507(65.1)	4375(64.9)	1132(66.1)	0.349
Women	2952(34.9)	2371(35.1)	581(33.9)	
Age, median (IQR)	72(24)	70(24)	81(20)	< 0.001
Length of hospital stay in days, median (IQR)	16(23)	16(23)	15(25)	0.529

*Abbreviations: IQR* Interquartile range

*P* value: Gender differences were analyzed using the chi-square test; age and length of hospital stay were analyzed using the Mann–Whitney U test

### DNR order rate, hospice care utilization rate, number of living days after DNR order, DOS after hospice enrollment, and late referral by diagnosis

Cancer patients had a significantly higher rate of DNR orders than non-cancer patients (94.9% vs. 73.5%, *p* < 0.001). Cancer decedents also had a significantly higher rate of hospice care utilization than non-cancer decedents (55.3% vs. 11.7%, *p* < 0.001).

Cancer decedents had fewer living days after DNR order than non-cancer decedents (median 7 days vs. 9 days, *p* < 0.001). Cancer patients had a longer DOS after hospice care enrollment than non-cancer decedents (median 13 days vs. 7 days, *p* < 0.001). Cancer decedents had a lower rate of late referral to hospice care than non-cancer decedents (33.9% vs. 53.2%, *p* < 0.001) (Table 2).

**Table 2:** Characteristics of do-not-resuscitate (DNR) order status and hospice care utilization by diagnoses: 2010–2015

Characteristics	Cancer	Non-cancer	*p*
DNR order, *n* (%)			< 0.001
No	347(5.1)	454(26.5)	
Yes	6399(94.9)	1259(73.5)	
Hospice care utilization, *n* (%)			< 0.001
No	3016(44.7)	1512(88.3)	
Yes	3730(55.3)	201(11.7)	
Number of living days after DNR order, median (IQR)	7(18)	9(27)	< 0.001
Length of hospice enrollment in days, median (IQR)	13(26)	7(13)	< 0.001
Late referral, %	33.9	53.2	< 0.001

*Abbreviations: IQR* Interquartile range

*P* value: DNR, hospice utilization, and late referral rates were analyzed using chi-square tests; number of living days after DNR order and length of hospice enrollment were analyzed using the Mann–Whitney U test

### Trends of DNR order rate, hospice care utilization rate, number of living days after DNR order, DOS after hospice enrollment, and late referral from 2010 to 2015

The cancer patient group showed a significantly increasing trend in hospice utilization rate but not in DNR order rate (*p* values for trend < 0.001 and *p* = 0.229, respectively). The non-cancer group showed a significantly increasing trend in the DNR order rate, although the increase was unsteady (*p* value for trend < 0.001). The rate of hospice utilization also increased gradually in the non-cancer group (*p* value for trend < 0.001).

The number of living days after DNR order increased significantly for both cancer and non-cancer patient groups (*p* value for trend = 0.001 and = 0.029, respectively). Neither cancer nor non-cancer groups showed significant trends for DOS after hospice care enrollment (*p* value for trend = 0.069 and = 0.134, respectively). The rates of late referral increased significantly in the cancer group but not in the non-cancer group (*p* value for trend < 0.001 vs. = 0.144, respectively) (Table 3).

**Table 3:** Trends in do-not-resuscitate (DNR) order and hospice care utilization variables: 2010 to 2015

Characteristics	Total	2010	2011	2012	2013	2014	2015	*p* for trend
DNR by disease, *n* (%)								
Cancer								0.229
No	347(5.1)	68(6.1)	59(5.3)	37(3.4)	47(4.0)	45(3.9)	91(8.3)	
Yes	6399(94.9)	1042(93.9)	1044(94.7)	1051(96.6)	1133(96.0)	1120(96.1)	1009(91.7)	
Non-cancer								< 0.001
No	454(26.5)	114(45.1)	83(27.8)	62(22.1)	55(19.9)	57(18.6)	83(27.9)	
Yes	1259(73.5)	139(54.9)	216(72.2)	219(77.9)	222(80.1)	249(81.4)	214(72.1)	
Hospice care utilization by disease, *n* (%)								
Cancer								< 0.001
No	3016(44.7)	574(51.7)	545(49.4)	479(44.0)	501(42.5)	491(42.1)	426(38.7)	
Yes	3730(55.3)	536(48.3)	558(50.6)	609(56.0)	679(57.5)	674(57.9)	674(61.3)	
Non-cancer								< 0.001
No	1512(88.3)	243(96.0)	274(91.6)	256(91.1)	250(90.3)	259(84.6)	230(77.4)	
Yes	201(11.7)	10(4.0)	25(8.4)	25(8.9)	27(9.7)	47(15.4)	67(22.6)	
Number of living days after DNR order, median (IQR)								
Cancer	7(18)	6(14)	7(17)	7(20)	8(20)	8(20)	9(19)	0.001
Non-cancer	9(27)	7(28)	8(23)	11(38)	8(22)	10(24)	11(31)	0.029
Length of hospice enrollment in days, median (IQR)								
Cancer	13(26)	13(27)	16(31)	14(28)	14(27)	12(25)	11(20)	0.069
Non-cancer	7(13)	14.5(158)	11(23)	7(31)	7(9)	6(16)	5(11)	0.134
Late referral, %								
Cancer	33.9	32.2	27.9	31.9	35.0	35.7	39.2	< 0.001
Non-cancer	53.2	30.0	48.0	52.0	51.9	55.3	58.2	0.144

*Abbreviations: IQR* Interquartile range

*P* for trend: DNR, hospice utilization, and late referral trend analyses were conducted using the non-parametric Jonckheere–Terpstra test; analysis of variance was performed to test for linear trends in number of living days after DNR order and length of hospice enrollment

## Discussion

There were several significant findings. DNR order rates, hospice care utilization rates, number of living days after DNR order, DOS after hospice care enrollment, and rates of late referral were significantly different between the cancer and non-cancer decedents. Furthermore, there were significantly increasing trends in hospice utilization for both cancer and non-cancer patient groups. However, there were no significant trends in the length of stay in hospice care for both groups, but there was a significant trend in the rate of late referral in the cancer group.

The results showed that cancer patients were more likely to have signed a DNR order and received hospice care before death. Cancer patients also had a longer mean length of stay in hospice care and a lower rate of late referrals to hospice care. These findings are similar to those of previous studies [14, 18]. There are several possible explanations for these results. First, cancer and non-cancer diseases show quite different disease trajectories. Typically, the trajectory for cancer is characterized as a short period of evident decline; the trajectory for organ failure features long-term limitations with intermittent serious episodes; and the trajectory for dementia consists of a prolonged decline [19]. It is thus substantially harder for clinicians to make precise prognoses for patients with organ failure or a disease associated with frailty than for cancer patients. In addition, the unexpected acute exacerbation of a non-cancer disease is less likely to be recognized as a terminal phase [14, 20].

Second, non-oncologists may be unsure how palliative and hospice care fits into their practice, partly because they receive less formal training and exposure to palliative care [14, 21, 22]. Clinicians may decide to provide palliative services or refer patients to hospice care teams based more on disease type (i.e., “malignant” vs. “benign”) than on the specific needs of individual patients and their families [4]. Third, discussion of death-related issues is taboo for some people in Asian cultures [15]. It may thus be difficult for clinicians to discuss DNR orders or hospice care with patients and their families owing to the unwelcome connotations of the terms “do not resuscitate” and “hospice” among non-cancer patients and their families.

However, non-cancer patients in this study had a greater number of living days after DNR order than cancer patients. This indicates that the non-cancer group patients tended to sign DNR orders earlier (relative to the date of death) than the cancer group patients. Boyd et al. found that, after controlling for the severity of illness as a confounding variable, older patients (≥ 75 years) were significantly more likely than younger patients to sign DNR orders [23]. Relatedly, a study by Crosby et al. revealed that older age was associated with early DNR orders [24]. In our study, non-cancer patents were significantly older than cancer patients (median age of 81 years vs. 70 years, *p* < 0.001).

For both the cancer and non-cancer patient groups, the rates of hospice care utilization increased significantly throughout the study period. DNR order rates also increased, albeit unsteadily, among the non-cancer group. Several factors may facilitate hospice care utilization and the use of DNR orders. Not only has hospice care in general been included in medical education in Taiwan [25], the Taiwanese government has also made various efforts to help people to understand and accept hospice care for terminal illness [26]. In addition, during the study period, Taiwan’s government made the second and third amendments (in 2011 and 2013, respectively) to the Hospice Palliative Care Act. These amendments allow the withdrawal of life-sustaining treatments from terminally ill patients following the agreement of one family member [16]. Additionally, the Taiwanese Department of Health has included hospice care utilization in its national hospital accreditation program since 2011 [26]. In addition to these policy changes and amendments, the provision of insurance reimbursement for hospice care for non-cancer patients since 2009 has also played an important role in encouraging hospice care utilization.

Although our findings showed no significant change in DOS after hospice enrollment among either terminal cancer patients or non-cancer patient groups from 2010 to 2015, the cancer group showed an increasing trend of late referrals to hospice care. The rate of late referrals also increased gradually, though non-significantly, in the non-cancer group. Because of their disease trajectories, the non-cancer diseases studied here can be considered chronic. Medical staff and family may thus exhaust all options (e.g., extracorporeal membrane oxygenation, mechanical ventilation, dialysis) to prolong the survival of terminal non-cancer patients. Studies of cancer patients have shown an increasing use of chemotherapies, such as target agents, and other aggressive forms of treatment (e.g., emergency room visits, intensive care unit admissions, inpatient days) during the end-of-life period [27, 28]. This trend is reflected in the present finding of an increase in late referrals to hospices and a decrease in the length of stay in hospice care.

Another important issue is the allotment of hospice resources. Awareness of the unmet hospice care needs of patients with terminal illness is growing in Taiwan. As the trend toward hospice utilization among non-cancer patients continues to increase, the relevant authorities need to pay more attention to such increased utilization, as it might compromise the ability of hospice care teams to provide care to cancer patients [3].

This study had several limitations. First, the data were collected from an urban tertiary center that includes a well-organized hospice care team. The results of the study may thus only be representative of hospitals at a similar level and with a similar degree of urbanization. Second, patients’ socioeconomic conditions (e.g., marital status, education level, residential area, and economic status) were not documented in the database. Therefore, we could not evaluate the associations between socioeconomic status and DNR orders and hospice care utilization rates. Third, only data for terminal patients who died in the hospital were included; data for those decedents who died at home, in nursing homes, or in other places were not included in the analyses. Fourth, many patients have multiple diseases and comorbidities. It is thus challenging to categorize patients strictly in terms of cancer or non-cancer diagnoses. In this study, our choice of the leading cause of death for a given patient was necessarily based on that patient’s medical chart. These limitations notwithstanding, this is the first study to compare DNR order and hospice care utilization rates for cancer patients and non-cancer terminal patients in Asia.

## Conclusions

DNR order rate, hospice care utilization rate, and length of hospice care enrollment were significantly higher among cancer patients than among non-cancer patients, whereas the number of living days after DNR order and the rate of late referral were significantly higher among the non-cancer decedents than among the cancer decedents. From 2010 to 2015, the cancer patient group showed significantly increasing trends in hospice utilization rate, number of living days after DNR order, and the rate of late referral. The non-cancer group showed significantly increasing trends in the rate of DNR order, hospice utilization rate, and the number of living days after DNR order. Further guidelines should be developed to help clinicians to promptly refer terminal cancer and non-cancer patients to hospice care. Furthermore, considering the lower hospice utilization rate and the growing need for hospice care among terminal non-cancer patients, policymakers should consider how to improve the relevant levels of professional care to enhance the accessibility and availability of hospice care in Taiwan.

## Abbreviations

DNR: Do not resuscitate
DOS: Duration of survival
NHI: National health insurance
DHPCD: Death and Hospice Palliative Care Database
IQR: Interquartile range
